# Comparative Genomics Reveals Pathogenicity-Related Loci in *Shewanella algae*

**DOI:** 10.1155/2020/9205197

**Published:** 2020-03-30

**Authors:** Jui-Hsing Wang, Guo-Cheng He, Yao-Ting Huang, Po-Yu Liu

**Affiliations:** ^1^Division of Infectious Disease, Department of Internal Medicine, Taichung Tzu Chi Hospital, Buddhist Tzu Chi Medical Foundation, Taichung 427, Taiwan; ^2^Department of Internal Medicine, School of Medicine, Tzu Chi University, Hualien 970, Taiwan; ^3^Department of Computer Science and Information Engineering, National Chung Cheng University, Chia-Yi 62102, Taiwan; ^4^Division of Infectious Diseases, Department of Internal Medicine, Taichung Veterans General Hospital, Taichung 40705, Taiwan; ^5^Rong Hsing Research Center for Translational Medicine, National Chung Hsing University, Taichung, Taiwan; ^6^Ph.D. Program in Translational Medicine, National Chung Hsing University, Taichung, Taiwan

## Abstract

*Shewanella algae* is an emerging marine zoonotic pathogen and accounts for considerable mortality and morbidity in compromised hosts. However, there is scarce literature related to the understanding of the genetic background of virulence determinants in *S. algae*. In this study, we aim to determine the occurrence of common virulence genes in *S. algae* using whole-genome sequence and comparative genomic analysis. Comparative genomics reveals putative-virulence genes related to bile resistance, chemotaxis, hemolysis, and motility. We detected the existence of *hlyA*, *hlyD*, and *hly*III involved in hemolysis. We also found chemotaxis gene cluster *cheYZA* operon and *cheW* gene. The results provide insights into the genetic basis underlying pathogenicity in *S. algae*.

## 1. Introduction

*Shewanella algae* is an emerging marine zoonotic pathogen. The organism was first classified in 1990 by Simidu et al. [1], emended by Nozue et al. [2], and described as a Gram-negative, motile bacillus, with hydrogen sulfide production, exhibiting hemolysis on sheep blood agar. *S. algae* is found in marine environments throughout the world and has been linked with both human and marine animal infections [3, 4]. Currently, there are at least three other *Shewanella* species found in clinical specimens and *S. algae* accounts for the majority of isolates from humans [5, 6]. *S. algae* has also been reported to cause diseases in marine animal, both wild and cultured [7–9]. However, there is scarce literature related to the understanding of the genetic background of virulence determinants in *S. algae*.

Marine ecosystem consists of a large variety of organisms that impact human health [10]. The advance of sequencing technology allows the identification of determinants in pathogenic microorganisms and has become an important approach to study the fundamental mechanisms of pathogenesis [11, 12]. Comparative genomics further enables the investigation of core elements of pathogenesis factors in great detail [13]. Recently, there have been attempts to use whole-genome sequencing in the study of marine pathogens [14]. Therefore, genomic comparison of the clinical *S. algae* isolates could provide clues for pathogenic or fitness determinants [15].

The aims of the study were to determine the occurrence of common virulence genes found in *S. algae* isolates from clinical setting using whole-genome sequence and comparative genomic analysis and to explore the relationship among the tested genomes.

## 2. Materials and Methods

### 2.1. Bacterial Strains, Media, and Growth Conditions

*S. algae* strains ACCC, YHL, and CHL were obtained from various clinical sources (Table 1). Glycerol stock of stored isolates was grown in trypticase soy agar with 5% sheep blood (Becton, Dickinson and Company, Franklin Lakes, NJ, USA) at 30°C for 24 hours. Single colonies were inoculated in tryptic soy broth (Becton, Dickinson and Company, Franklin Lakes, NJ). The isolates were preliminarily identified using 16S rRNA gene sequencing and matrix-assisted laser desorption ionization-time of flight mass spectrometry (bioMérieux, Marcy l'Etoile, France). A part of 16S rRNA gene was amplified using the primers of B27F (5′-AGAGTTTGATCCTGGCTCAG-3′) and U1492R (5′-GGTTACCTTGTTACGACTT-3′) [9, 16]. The nucleotide sequences were aligned, and BLAST search was performed against the GenBank database of the National Center for Biotechnology Information (NCBI) [17].

### 2.2. Genome Sequencing and Assembly

Nucleic acids were extracted from overnight culture using the QIAGEN Genomic-tip 100/G kit and the Genomic DNA Buffer Set (QIAGEN, Paisley, UK) according to the manufacturer's protocol. The DNA concentrations were measured by Qubit dsDNA HS Assay kit using Qubit 2.0 fluorometer (Life Technologies, Carlsbad, CA, USA). The DNA sample was sheared, in a microTUBE using Covaris S2 (Covaris, Woburn, MA, USA), into the desired size fragment of the library. The indexed PCR-free library preparation was performed using multiplexed high-throughput sequencing TruSeq DNA Sample Preparation Kit (Illumina) with 2 *μ*g of DNA on the basis of the manufacturer's introduction. Genome sequencing was performed using paired-end 250 bp sequencing on the Illumina MiSeq platform (Illumina, Inc., San Diego, CA). Raw sequence files were artifact-filtered and trimmed with DUK (http://duk.sourceforge.net/) and FASTX-toolkit fastx_trimmer (https://github.com/agordon/fastx_toolkit), respectively. Assembly was performed with a hybrid approach by ALLPATHS, version R46652 and Velvet version 1.2.07.

### 2.3. Public Genome Download

Genome sequence of human isolated *S. algae* MARS 14 was retrieved from the NCBI Genome website (https://www.ncbi.nlm.nih.gov/assembly/GCF_000947195.1/).

### 2.4. Phylogenetic Analysis Based on Whole-Genome Sequences

Genome-based phylogenic analysis was performed using pairwise comparison of average nucleotide identity. The whole-genome average nucleotide identity (ANI) was calculated with the use of a modified algorithm [18]. Phylogenetic trees were visualized using MEGA7.

### 2.5. Annotation and Comparative Genomics

The annotation was performed using the NCBI Prokaryotic Genome Annotation Pipeline (PGAP) [19] and the DOE-JGI Microbial Genome Annotation Pipeline version 4.10.5 [20]. The prediction was done using Glimmer 3.02 [21]. The nontranslated genes were predicted by tRNAscan-SE [22], RNAmmer [23], and RFAM [24]. Functional classification of the predicted genes was carried out using RPSBLAST program v. 2.2.15 [25]. Analysis of the functional annotation was further performed using the Integrated Microbial Genomes & Microbiomes system v.5.0 [26] and the Pathosystems Resource Integration Center [27]. CDS count for these strains was derived. Comparative genome analysis was performed using EDGAR platform (http://edgar.computational.bio) [28]. The core genome and the singletons for the 4 related *S*. *algae* genomes were generated for Prokka-annotated genomes using EDGAR (http://edgar.computational.bio). We compared the *S. algae* genomes using the MUMmer software package [29] together with the Circos visualization engine [30].

## 3. Results

### 3.1. Genome Sequencing and Assembly

The genomic sequencing consisted of 250 bp paired-end reads, yielding approximately 0.88 Gbp to 1.24 Gbp for each isolate. The de novo assembly of genome sequence data revealed that the number of contigs (>200 bp) varied from 27 to 74 for each genome. The maximum contig size among the genomes was 976,090 bp aligned to YHL. The GC content ranged from 52.96% for CHL to 53.08% for ACCC. Table 1 shows the descriptive statistics of the genomic characteristics for the strains in this study. The sequence data were publicly available in NCBI SRA database (accession number: ACCC [LVCY00000000.1], CHL [LVDF00000000.1], and YHL [LVDU00000000.1]).

### 3.2. Genome-Based Phylogenetic Analysis

**Figure 1:**
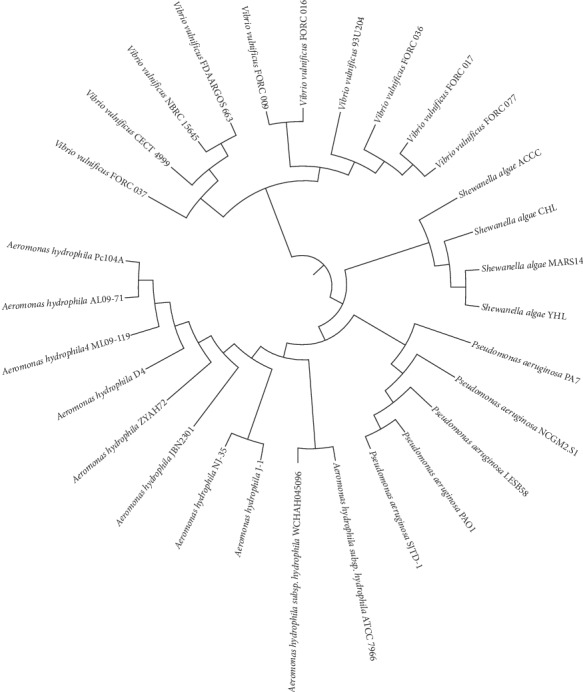
Whole-genome phylogeny of *S. algae* in the study.

The average nucleotide identity (ANI) was calculated and revealed that tested *S. algae* strains were identical in terms of nucleotide sequences, as shown in Figure 1.

### 3.3. Comparative Genomics

**Figure 2:**
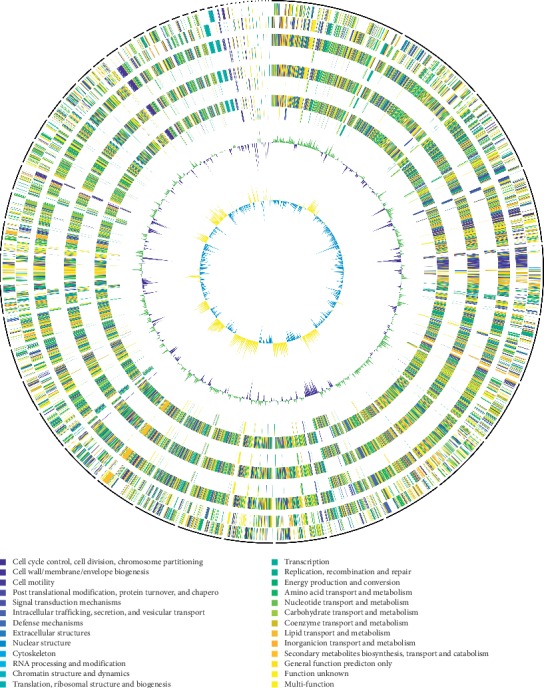
Circular genomes representation map and genome comparison of *Shewanella algae* (CHL, ACCC, MARS 14, and YHL). Predicted coding sequences (CDSs) are assigned various colors with respect to cellular functions. Circles show, from the outermost to the innermost, (1) DNA coordinates; (2, 3) function-based color-coded mapping of the CDSs predicted on the forward and reverse strands of the *S. algae* CHL genome, respectively; (4) orthologous CDSs shared between *S. algae* CHL and *S. algae* ACCC; (5) *S. algae* CHL-specific CDSs, compared with *S. algae* ACCC; (6) orthologous CDSs shared between *S. algae* CHL and *S. algae* MARS 14; (7) *S. algae* CHL-specific CDSs, compared with *S. algae* MARS 14; (8) orthologous CDSs shared between *S. algae* CHL and *S. algae* YHL; (9) *S. algae* CHL-specific CDSs, compared with *S. algae* YHL; (10) GC plot with regions above and below average in green and violet; (11) GC skew showing regions above and below average in yellow and light blue. This figure was plotted in Scalable Vector Graphics format via an in-house script, which calculates the radius and ribbon width according to the BLAST alignments and adds colors by COG classification of all orthogonal genes.

**Figure 3:**
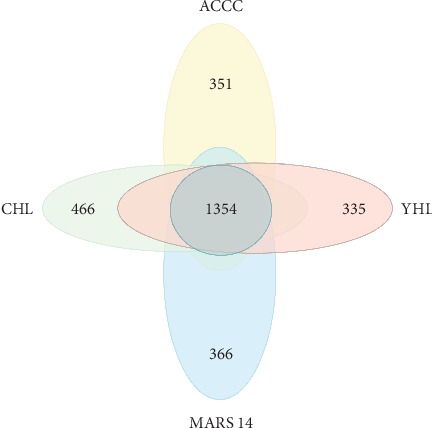
Comparison of the gene contents of the *Shewanella algae* in this study, Venn diagram showing the numbers of conserved and strain-specific coding sequences (CDSs).

**Figure 4:**
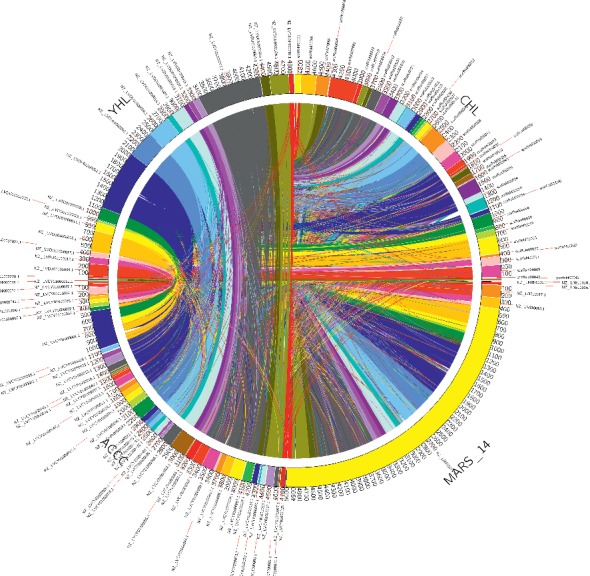
Genomes mapping between strains in the study. Each colored arc indicates an orthologous match between two species. The color segments in the outer circle are randomly displayed and do not correspond to a particular scheme. A minimum seed match size of 500 bp was used.

We constructed a pan-genome dataset using whole-genome sequence of sequenced *S. algae* strains.  Figure 2 shows orthologous genes shared among strains and depicts the position and color-coded function of the *S. algae* genes. The numbers of orthologous and strain-specific unique genes are shown in the Venn diagram. Core genome for the *S. algae* strains consists of 1354 coding sequences (Figure 3). The set of unique genes harbored by each strain varies from 335 for *S. algae* YHL to 466 for *S. algae* CHL. Following genome map construction, we conducted genome mapping among the *S. algae* strains in the study. In this comparison, colored arcs indicate regions of high similarity as revealed by the NUCmer script from the MUMmer software package. As shown in Figure 4, the alignment revealed an obvious syntenic relationship in these strains.

### 3.4. Analysis of Putative-Virulence-Related Genes

As illustrated in Table 2, genes encoded *exbBD*, *galU*, and *htpB* are shared with *S*. *algae* genomes. Heat shock protein gene *clpP* and hemolysis homologous genes, *hlyA*, *hlyD*, *hly*III, and *tolC*, were found in each *S*. *algae* genome. Gene cluster *cheYZA* operon and *cheW* involved in chemotaxis were detected in all tested *S*. *algae*. Flagellar gene operons are present in all tested *S*. *algae* genome.

## 4. Discussion

*S. algae* has become an emerging marine zoonotic pathogen world-wide [5]. The spectrum of *S*. *algae* infection is broad with considerable morbidity and mortality in compromised hosts [31, 32]. Thus, understanding genomic characterization of *S*. *algae* is important for determining molecular epidemiology, understanding its pathogenesis, identifying specific biomarkers, tracing evolution of these strains, and developing control strategy of these pathogens in host reservoirs. In this study, we investigated the core genetic structure underlying *S*. *algae* virulence. The pathogenicity and distribution patterns of the *S*. *algae* strains extended our understanding of their pathogenic potential.

Previous attempts have been made to report the basic features of the genome of *S*. *algae* from various sources [33, 34]. In the present study, we used comparative genomics to analyze chromosomal sequence of four isolates to determine the common genetic content and organization, unique virulence attributes, and evolutionary relationship with other strains. Whole-genome sequence analysis of *S*. *algae* detected the presence of chemotaxis gene cluster *cheYZA* operon that is conserved in the chemotactic bacteria [35]. Chemotaxis is a directed motility in response to concentration gradients of signals. The *cheA* was demonstrated to be essential for chemotaxis using a two-component pathway [36]. In brief, CheA phosphorylates *cheY* and then is dephosphorylated by the phosphatase *cheZ* [37]. Previous studies revealed that CheW and CheA share structural homology and bind to the same site on chemoreceptors [37]. CheW is essential to the activation of CheA and the formation of CheA-CheW complex [38]. Owing to the wide range of *S*. *algae* habitats, the drivers of its chemotaxis could be very diverse. Previous studies have demonstrated that pathogenic bacteria use chemotaxis to localize reservoirs. Further study would be needed to identify the microenvironments suit for *S*. *algae* and the trigger of its chemotaxis.

Biliary tract infection is main manifestation of *S*. *algae* infection, and bile resistance has been noted in pathogenic strains [31]. In the study we also identified genes associated with bile adaption. The *exbBD* gene encodes Ton energy transduction system implicated in the response to bile [39, 40]. We also detected *galU*, *htpB*, and *wecA* involved in bile resistance [41–43]. The results support an earlier genomic study suggesting a common mechanism of bile resistance in *Shewanella*.

Motility is one characteristic of *S*. *algae* [3]. We identified series of flagellar gene operons in *S*. *algae* genomes. These flagellar systems are unique and require more study regarding the evolution and organization. Hemolysis is a main pathogenic feature in *S*. *algae* [44]. The gene *hlyA* encodes RTX pore-forming toxin *α*-hemolysin, which alters membrane permeability and causes cell lysis in a variety of human and animal hosts [45].

## 5. Conclusions

In conclusion, this is one of the few studies tracking genetic background of putative virulence-related genes in *S*. *algae*. Although the number of strains was limited, we highlight the unique characteristics of core virulence determinants in these strains, as a high level of genomic conservation.

## Figures and Tables

**Table 1:** Strains and genomic features of *S. algae* strains in this study.

Strain	Isolation source	Geographic origin	Genome assembly status	Genome coverage	Genome size (bp)	GC content (%)	CDSs	Pseudogenes	rRNA operons	tRNAs
CHL	Bile	Taiwan	Scaffold	243.0x	4,888,589	52.96	4,281	122	6, 5, 2 (5S, 16S, 23S)	88
YHL	Wound	Taiwan	Scaffold	257.0x	4,850,439	53.00	4,212	71	6, 5, 2 (5S, 16S, 23S)	86
ACCC	Bile	Taiwan	Scaffold	186.0x	4,744,804	53.08	4,223	143	4, 4 (5S, 16S)	91
MARS 14	Lung	France	Scaffold	91.0x	5,005,849	52.90	4,347	90	6, 3, 3 (5S, 16S, 23S)	104

**Table 2:** Virulence genes shared with *S. algae* strains in this study.

Gene	locus_tag	Length	locus_tag	Length	locus_tag	Length	locus_tag	Length
Strains	MARS 14		YHL		CHL		ACCC	

*hlyA*	BN1227_RS19795	443	AYI97_RS17645	443	AYI82_RS07480	443	AYI77_RS13890	443

*hlyD*	BN1227_RS18765	352	AYI97_RS03440	352	AYI82_RS00925	352	AYI77_RS05040	352
	BN1227_RS19395	349	AYI97_RS04065	349	AYI82_RS01570	349	AYI77_RS08410	349
	BN1227_RS08585	314	AYI97_RS05045	314	AYI82_RS17560	314	AYI77_RS14620	314

*hlyIII*	BN1227_RS10295	226	AYI97_RS09385	226	AYI82_RS06545	226	AYI77_RS04320	226

*tolC*	BN1227_RS02290	424	AYI97_RS03690 AYI97_RS12455 AYI97_RS17535 AYI97_RS18425	466 438 467 491	AYI82_RS01185 AYI82_RS03510 AYI82_RS07370	466 438 467	AYI77_RS01075 AYI77_RS01845 AYI77_RS02720 AYI77_RS04785 AYI77_RS13995	491
	BN1227_RS02895	438						438
	BN1227_RS03705	491						424
	BN1227_RS12395	438						466
	BN1227_RS19025	466						467
	BN1227_RS19685	467						

*htpB (groL)*	BN1227_RS18535	546	AYI97_RS03170	546	AYI82_RS00700	546	AYI77_RS05265	546

*galU*	BN1227_RS14240	303	AYI97_RS19990	303	AYI82_RS13425	303	AYI77_RS03360	303
					AYI82_RS18495	294	AYI77_RS16730	294

*exbB*	BN1227_RS13275	175	AYI97_RS02650	238	AYI82_RS00200	238	AYI77_RS07050	175
	BN1227_RS13280	451	AYI97_RS04225	164	AYI82_RS01760	164	AYI77_RS07055	451
	BN1227_RS17925	238	AYI97_RS14790	175	AYI82_RS21410	175	AYI77_RS08255	164
	BN1227_RS19555	164	AYI97_RS14795	451	AYI82_RS21415	451	AYI77_RS08875	238

*exbD*	BN1227_RS13270	134	AYI97_RS02655	135	AYI82_RS00205	135	AYI77_RS07045	134
	BN1227_RS17930	135	AYI97_RS04230	135	AYI82_RS01765	135	AYI77_RS08250	135
	BN1227_RS19560	135	AYI97_RS14785	134	AYI82_RS21405	134	AYI77_RS08870	135

*cheY*	BN1227_RS07095	127	AYI97_RS06385	127	AYI82_RS05630	127	AYI77_RS20450	127

*cheZ*	BN1227_RS07100	245	AYI97_RS06380	245	AYI82_RS05625	245	AYI77_RS20455	245

*cheA*	BN1227_RS01115	701	AYI97_RS06375	776	AYI82_RS05620	770	AYI77_RS12010	696
	BN1227_RS07105	776	AYI97_RS16805	696	AYI82_RS09360	701	AYI77_RS20925	754

*cheW*	BN1227_RS07130	164	AYI97_RS06350	164	AYI82_RS05595	164	AYI77_RS20950	164
	BN1227_RS01120	183	AYI97_RS16800	183	AYI82_RS05600	336	AYI77_RS12015	183
	BN1227_RS07125	336	AYI97_RS06355	335	AYI82_RS09355	183	AYI77_RS20945	336

*clpP*	BN1227_RS08465	202	AYI97_RS05170	202	AYI82_RS17685	202	AYI77_RS14495	202

*FlgA*	BN1227_RS06885	235	AYI97_RS06595	235	AYI82_RS05050	248	AYI77_RS09380	248
	BN1227_RS21260	248	AYI97_RS14310	248	AYI82_RS05840	235	AYI77_RS20850	235

*FlgB*	BN1227_RS06900	132	AYI97_RS06580	132	AYI82_RS05045	116	AYI77_RS09385	116
	BN1227_RS21255	116	AYI97_RS14305	116	AYI82_RS05825	132	AYI77_RS20835	132

*FlgC*	BN1227_RS06905	138	AYI97_RS06575	138	AYI82_RS05040	136	AYI77_RS09390	136
	BN1227_RS21250	136	AYI97_RS14300	136	AYI82_RS05820	138	AYI77_RS20830	138

*FlgD*	BN1227_RS21245	221	AYI97_RS06570	227	AYI82_RS05035	221	AYI77_RS09395	221
			AYI97_RS14295	221	AYI82_RS05815	227	AYI77_RS20825	227

*FlgE*	BN1227_RS06915	453	AYI97_RS06565	453	AYI82_RS05810	453	AYI77_RS20820	453

*FlgF*	BN1227_RS06920	247	AYI97_RS06560	247	AYI82_RS05805	247	AYI77_RS20815	247

*FlgG*	BN1227_RS06925	262	AYI97_RS06555	262	AYI82_RS05020	261	AYI77_RS09410	261
	BN1227_RS21230	261	AYI97_RS14280	261	AYI82_RS05800	262	AYI77_RS20810	262

*FlgH*	BN1227_RS06930	224	AYI97_RS06550	363	AYI82_RS05015	223	AYI77_RS09415	223
	BN1227_RS21225	223	AYI97_RS14275	224 223	AYI82_RS05795	224	AYI77_RS20805	224

*FlgI*	BN1227_RS06935	363	AYI97_RS06545	363	AYI82_RS05010	373	AYI77_RS09420	359
	BN1227_RS21220	373	AYI97_RS14270	373	AYI82_RS05790	363	AYI77_RS20800	363

*FlgJ*	BN1227_RS06940	336	AYI97_RS06540	336	AYI82_RS05785	336	AYI77_RS20795	336

*FlgK*	BN1227_RS06945	641	AYI97_RS06535	641	AYI82_RS05000	456	AYI77_RS09430	456
	BN1227_RS21210	456	AYI97_RS14260	456	AYI82_RS05780	641	AYI77_RS20790	641

*FlgL*	BN1227_RS06950	401	AYI97_RS06530	401	AYI82_RS05775	401	AYI77_RS20785	

*FlgM*	BN1227_RS06880	106	AYI97_RS06600	106	AYI82_RS05055	94	AYI77_RS09375	94
	BN1227_RS21265	94	AYI97_RS14315	94	AYI82_RS05845	106	AYI77_RS20855	106

*FlgN*	BN1227_RS06875	143	AYI97_RS06605	143	AYI82_RS05060	171	AYI77_RS09370	171
					AYI82_RS05850	143	AYI77_RS20860	143

*FlgP*	BN1227_RS06870	155	AYI97_RS06610	155	AYI82_RS05855	155	AYI77_RS20865	155

*FlgT*	BN1227_RS06860	385	AYI97_RS06620	385	AYI82_RS05865	385	AYI77_RS20875	385

FliA	BN1227_RS07090	239	AYI97_RS06390	239	AYI82_RS04955	236	AYI77_RS20445	239
	BN1227_RS21165	236	AYI97_RS14215	236	AYI82_RS05635	239	AYI77_RS09475	236

FliD	BN1227_RS06970	456	AYI97_RS06510	456	AYI82_RS04980	445	AYI77_RS20325	451
	BN1227_RS21190	445	AYI97_RS14240	445	AYI82_RS05755	456		

FliE	BN1227_RS07000	110	AYI97_RS06480	110	AYI82_RS05090	111	AYI77_RS09340	111
	BN1227_RS21300	111	AYI97_RS14350	111	AYI82_RS05725	110	AYI77_RS20355	110

FliF	BN1227_RS07005	569	AYI97_RS06475	569	AYI82_RS05085	555	AYI77_RS09345	555
	BN1227_RS21295	555	AYI97_RS14345	555	AYI82_RS05720	569	AYI77_RS20360	569

FliG	BN1227_RS07010	347	AYI97_RS06470	347	AYI82_RS05080	328	AYI77_RS09350	324
	BN1227_RS21290	328	AYI97_RS14340	328	AYI82_RS05715	347	AYI77_RS20365	347

FliH	BN1227_RS07015	322	AYI97_RS06465	324	AYI82_RS05710	324	AYI77_RS20370	324

FliI	BN1227_RS07020	446	AYI97_RS06460	446	AYI82_RS05070	441	AYI77_RS09360	441
	BN1227_RS21280	441	AYI97_RS14330	441	AYI82_RS05705	446	AYI77_RS20375	446

FliJ	BN1227_RS07025	149	AYI97_RS06455	149	AYI82_RS05700	149	AYI77_RS20380	149

FliL	BN1227_RS00740	135	AYI97_RS06445	174	AYI82_RS04960	145	AYI77_RS11650	135
	BN1227_RS07035	174	AYI97_RS14220	145	AYI82_RS05690	174	AYI77_RS20390	174
	BN1227_RS21170	145	AYI97_RS17155	135	AYI82_RS09710	135		

FliM	BN1227_RS07040	342	AYI97_RS06440	342	AYI82_RS05685	342	AYI77_RS18030	238
	BN1227_RS21315	300	AYI97_RS14365	300			AYI77_RS20395	342

FliN	BN1227_RS07045	126	AYI97_RS06435	126	AYI82_RS05110	114	AYI77_RS18025	114
	BN1227_RS21320	114	AYI97_RS14370	114	AYI82_RS05680	126	AYI77_RS20400	126

FliO	BN1227_RS07050	119	AYI97_RS06430	119	AYI82_RS05675	119	AYI77_RS20405	119

FliP	BN1227_RS07055	247	AYI97_RS06425	247	AYI82_RS05115	265	AYI77_RS18020	265
	BN1227_RS21325	265	AYI97_RS14375	265	AYI82_RS05670	247	AYI77_RS20410	247

FliQ	BN1227_RS07060	89	AYI97_RS06420	89	AYI82_RS05120	89	AYI77_RS18015	89
	BN1227_RS21330	89	AYI97_RS14380	89	AYI82_RS05665	89	AYI77_RS20415	89

FliR	BN1227_RS07065	265	AYI97_RS06415	265	AYI82_RS05125	259	AYI77_RS18010	259
	BN1227_RS21335	259	AYI97_RS14385	259	AYI82_RS05660	265	AYI77_RS20420	265

FliS	BN1227_RS06980	136	AYI97_RS06500	136	AYI82_RS04975	126	AYI77_RS09455	126
	BN1227_RS21185	126	AYI97_RS14235	126	AYI82_RS05745	136	AYI77_RS20335	136

flhA	BN1227_RS21345	692	AYI97_RS14395	692	AYI82_RS05135	692	AYI77_RS18000	692
	BN1227_RS07075	701	AYI97_RS06405	701	AYI82_RS05650	701	AYI77_RS20430	701

flhB	BN1227_RS07140	105	AYI97_RS06340	105	AYI82_RS05585	105	AYI77_RS20960	105
	BN1227_RS21340	376	AYI97_RS14390	376	AYI82_RS05130	376	AYI77_RS18005	376
	BN1227_RS07070	378	AYI97_RS06410	378	AYI82_RS05655	378	AYI77_RS20425	378

flhF	BN1227_RS07080	458	AYI97_RS06400	458	AYI82_RS05645	458	AYI77_RS20435	458

## Data Availability

The sequence data are publicly available in NCBI SRA database (accession number: ACCC [LVCY00000000.1], CHL [LVDF00000000.1], and YHL [LVDU00000000.1]).
